# Generation and Characterization of Human iPSC-Derived Astrocytes with Potential for Modeling X-Linked Adrenoleukodystrophy Phenotypes

**DOI:** 10.3390/ijms26041576

**Published:** 2025-02-13

**Authors:** Navtej Kaur, Jaspreet Singh

**Affiliations:** 1 Department of Neurology, Henry Ford Hospital, Detroit, MI 48202, USA; nkaur4@hfhs.org; 2 Department of Physiology, Michigan State University, Lansing, MI 48824, USA

**Keywords:** ABCD1, induced pluripotent stem cells, astrocytes, X-ALD, mitochondria, cytokines

## Abstract

X-adrenoleukodystrophy (X-ALD) is a peroxisomal metabolic disorder caused by mutations in the ABCD1 gene encoding the peroxisomal ABC transporter adrenoleukodystrophy protein (ALDP). Similar mutations in ABCD1 may result in a spectrum of phenotypes in males with slow progressing adrenomyeloneuropathy (AMN) and fatal cerebral adrenoleukodystrophy (cALD) dominating most cases. Mouse models of X-ALD do not capture the phenotype differences and an appropriate model to investigate the mechanism of disease onset and progress remains a critical need. Here, we generated induced pluripotent stem cell (iPSC) lines from skin fibroblasts of two each of apparently healthy control, AMN, and cALD patients with non-integrating mRNA-based reprogramming. iPSC lines expanded normally and expressed pluripotency markers Oct4, SOX2, NANOG, SSEA, and TRA-1–60. Expression of markers SOX17, Brachyury, Desmin, OXT2, and beta tubulin III demonstrated the ability of the iPSCs to differentiate into all three germ layers. iPSC-derived lines from CTL, AMN, and cALD male patients were differentiated into astrocytes. Differentiated AMN and cALD astrocytes lacked ABCD1 expression and accumulated saturated very long chain fatty acids (VLCFAs), a hallmark of X-ALD, and demonstrated differential mitochondrial bioenergetics, cytokine gene expression, and differences in STAT3 and AMPK signaling between AMN and cALD astrocytes. These patient astrocytes provide disease-relevant tools to investigate the mechanism of differential neuroinflammatory response in X-ALD and will be valuable cell models for testing new therapeutics.

## 1. Introduction

The most common peroxisomal disorder affecting males at early ages, adrenoleukodystrophy (ALD), results from deletion/mutation in the ABCD1 gene, leading to an absent or non-functioning adrenoleukodystrophy protein (ALDP) [1,2]. This defect causes an accumulation of very long chain fatty acids (VLCFAs) in tissues and plasma via inhibition of peroxisomal β-oxidation [1,3,4]. The accumulation of VLCFA is the hallmark of X-ALD disease. Two major clinical variants exist: cerebral ALD (cALD) and adrenomyeloneuropathy (AMN). Though caused by the same or a similar mutation or deletion, cALD is biochemically associated with redox alterations, inflammation, and subsequent loss of myelin/oligodendrocytes [4]. cALD is often fatal in childhood, whereas AMN patients live to adulthood with mild involvement of the peripheral nervous system [1,4,5,6].

Unlike human cALD, Abcd1-knock out (KO) mice do not show cerebral pathology [7,8,9]. However, they present AMN-like symptoms and show imbalance in antioxidant systems with increasing age [10,11]. The mechanisms that cause the spontaneous progression of disease from relatively mild AMN to fatal cALD remain unclear. We and others have documented a role for Abcd1-silenced astrocytes in X-ALD neuroinflammatory response [12,13,14]. Mice astrocytes silenced for Abcd1 and Abcd2 produced a spontaneous inflammatory phenotype [13]. We reported inflammatory response in unstimulated Abcd1-KO mice astrocytes in vitro silenced for AMPKα1 [14]. Mouse astrocytes, however, could not be used to model the differential inflammatory response seen in human AMN and cALD phenotypes.

Induced pluripotent stem cells (iPSCs) have become an attractive tool for in vitro disease modeling since they can give rise to any cell of the body. Skin fibroblasts were the first human cells to be reprogrammed to iPSCs due to their ease of availability and growth in culture [15,16]. In patient astrocytes obtained by directed differentiation of iPSCs derived from skin fibroblasts of apparently healthy control (CTL), AMN, and cALD males, we documented differential mitochondrial dysfunction, oxidative stress response, and neuroinflammatory cytokine profile [17]. In this work we generated iPSC-derived astrocytes from two additional male patients each of CTL, AMN, and cALD phenotypes. iPSCs were in turn obtained by reprogramming of human skin fibroblasts from CTL, AMN, and cALD. iPSC-derived astrocytes were further characterized for mitochondrial function, differential cytokine response, and STAT3 and AMPK signaling pathway.

## 2. Results

### 2.1. CTL, AMN, and cALD Patient Fibroblast-Derived iPSCs Are Positive for AP and Expressed Pluripotency Markers

**Figure 1:**
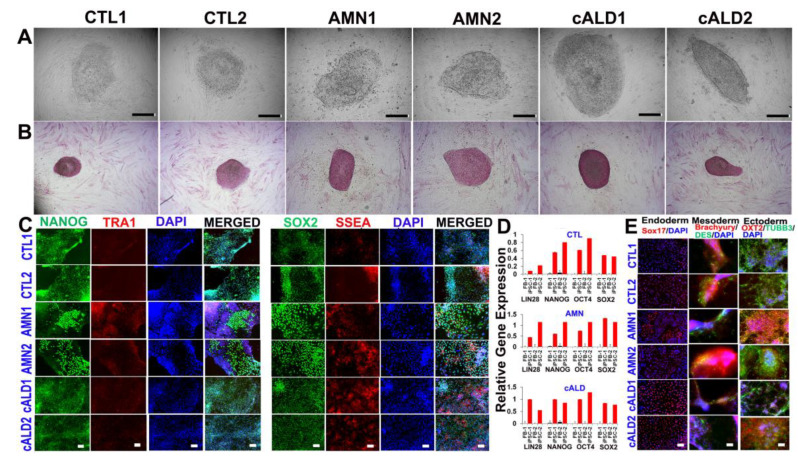
Generation of iPSCs from patient-derived skin fibroblasts. (**A**) Representative phase contrast images of CTL, AMN, and cALD iPSCs. (**B**) Representative alkaline-phosphatase-stained images of CTL, AMN, and cALD iPSCs. (**C**) Immunostaining for pluripotency markers: NANOG, TRA1–60, SOX2, and SSEA. Nuclei are stained with DAPI (blue). (**D**) RT-qPCR quantification of pluripotency markers (ratio against L27) in CTL, AMN iPSCs (*n* = 3). (**E**) Representative immunocytochemistry showed iPSC-derived cells positive for markers of three germ layers following differentiation: endoderm (SOX17), mesoderm (BRACHYURY and DESMIN), ectoderm (OXT2 and TUBB3). AMN: Adrenomyeloneuropathy; cALD: Cerebral adrenoleukodystrophy; CTL: Control; iPSC: Induced pluripotent stem cell. Scale: 100 µM (**A**,**B**) and 50 µM (**C**,**E**).

CTL, AMN, and cALD human dermal fibroblasts were transfected with non-modified RNA (NM-RNA) containing OCT4, SOX2, KLF4, cMYC, NANOG, and LIN28 reprogramming factors and NM-miRNA as per the manufacturer’s protocol (ReproCell technologies). Daily overnight transfections were performed for four consecutive days and cells were cultured in a hypoxic environment with 5% oxygen. The earliest emergence of iPSC colonies was observed ten days following the last transfection. Colonies had a typical round and flat iPSC colony structure with a defined colony boundary separating it from the fibroblasts (Figure 1A). Pluripotency was established by checking for alkaline phosphatase activity (Figure 1B) and alkaline phosphatase live staining (Supplementary Figure S1). The iPSC clones showed typical morphology and normal karyotype at passage 5, as assessed by the KaryoStat assay (Supplementary Figure S2).

### 2.2. Expression of Pluripotency Markers in CTL, AMN, and cALD Fibroblast-Derived iPSCs

iPSC colonies were characterized for pluripotency markers after five passages. Fluorescent staining for pluripotent markers documented significant expression of NANOG, TRA-1–60, SOX2, and SSEA in iPSC colonies from CTL, AMN, and cALD (Figure 1C). RT-qPCR evaluation of CTL, AMN, and cALD patient fibroblasts and the corresponding iPSCs showed significantly higher expression of LIN28, NANOG, OCT4, and SOX2 in iPSCs compared to fibroblasts (Figure 1D).

### 2.3. Differentiation of iPSC Colonies into Germ Layers

Functional activities of iPSCs were determined by evaluating their ability to differentiate into three germ layers (Human pluripotent stem cell functional identification kit, R&D systems, Cat#SC027B). Immunofluorescent analysis of CTL, AMN, and cALD cells’ disaggregated form embryoid bodies revealed various cell derivatives expressing ectodermal (OXT2 and β-III-tubulin), mesodermal (brachyury and desmin), and endodermal (SOX17) markers (Figure 1E). Thus, the obtained human iPSCs possess a broad differentiation potential in vitro.

### 2.4. Astrocyte Differentiation of CTL, AMN, and cALD iPSC Colonies

**Figure 2:**
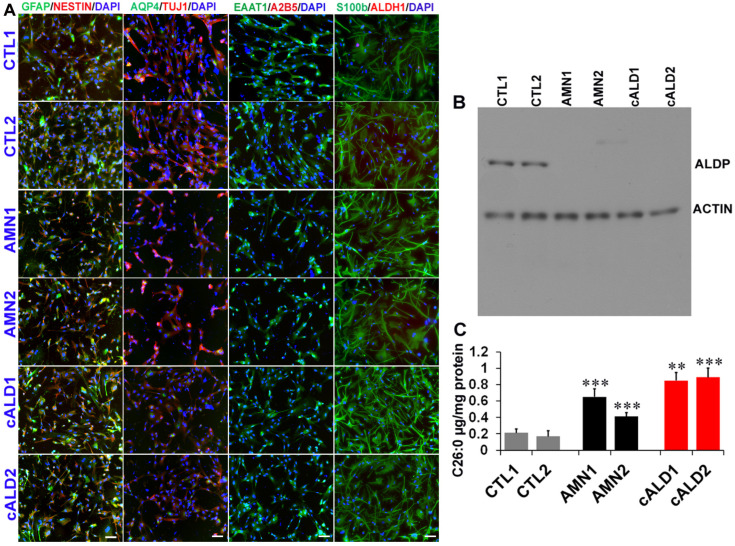
Differentiation of iPSCs into astrocytes. (**A**) Immunostaining for astrocyte markers: GFAP, AQP4, EAAT1, and S100β. Nuclei are stained with DAPI (blue). (**B**) Representative Western blot for ABCD1 and β-actin in CTL, AMN iPSCs. (**C**) C26:0 VLCFA levels measured by LC-MS in CTL, AMN, and cALD astrocytes. AMN: Adrenomyeloneuropathy; cALD: Cerebral adrenoleukodystrophy; CTL: Control; iPSC: Induced pluripotent stem cell. ** *p* < 0.01; *** *p* < 0.001; Scale: 50 µM.

Monolayers of cells were plated on Matrigel-coated plates in neural induction medium with SMAD inhibitor (SMADi) for 12–15 days to generate neural precursor cells using a STEMdiff™ SMADi Neural Induction kit (STEMCELL Technologies, Kent, WA, USA). SMADi inhibits the expression of pluripotency genes and suppresses differentiation in the mesodermal and ectodermal directions. Neural precursors were driven to an astrocytic lineage by further culturing the cells on Matrigel-coated plates in astrocyte differentiation media for 18–20 days with media change every 2–3 days and passage every 6–8 days (STEMdiff^TM^ Astrocyte differentiation kit, Stemcell Technologies). After 20–21 days on astrocyte differentiation medium, cells were incubated further in astrocyte maturation medium for 25–30 days with media changes every 2–3 days (STEMdiff^TM^ Astrocyte maturation kit. STEMCELL Technologies). The cells were passaged every 6–8 days. After at least three passages, mature astrocytes were identified by immunofluorescence staining for mature glial cell markers, including GFAP, aquaporin 4, S100β, and EAAT1 (Figure 2A). Cells were negative for A2B5. ABCD1 mutation did not seem to affect the differentiation potential of iPSCs to astrocytes.

### 2.5. ABCD1 Expression and VLCFA Levels in CTL, AMN, and cALD iPSC-Derived Astrocytes

ABCD1 mutation is associated with loss of ALDP protein in AMN and cALD. We next investigated the status of ALDP in iPSC-derived CTL, AMN, and cALD astrocytes by Western blotting. AMN and cALD iPSC-derived astrocytes exhibited loss of ALDP (Figure 2B). Loss of ALDP is associated with decreased β-oxidation and resultant VLCFA accumulation in X-ALD. We measured VLCFA (C26:0) levels in control, AMN, and cALD iPSC-derived astrocytes. Absolute levels of C26:0 were significantly increased in AMN astrocytes compared to CTL astrocytes (Figure 2C). The C26:0 levels in cALD were further increased significantly compared to AMN astrocytes (Figure 2C).

### 2.6. Mycoplasma Detection in CTL, AMN, and cALD iPSC-Derived Cells

Quality control of iPSC-derived cells was performed by nucleic acid amplification to detect mycoplasma species. No mycoplasma contamination was detected by PCR in any of the six CTL, AMN, and cALD iPSC lines (Supplementary Figure S3).

### 2.7. Glycolytic Rate Is Significantly Increased in cALD iPSC-Derived Astrocytes Compared to AMN Astrocytes

**Figure 3:**
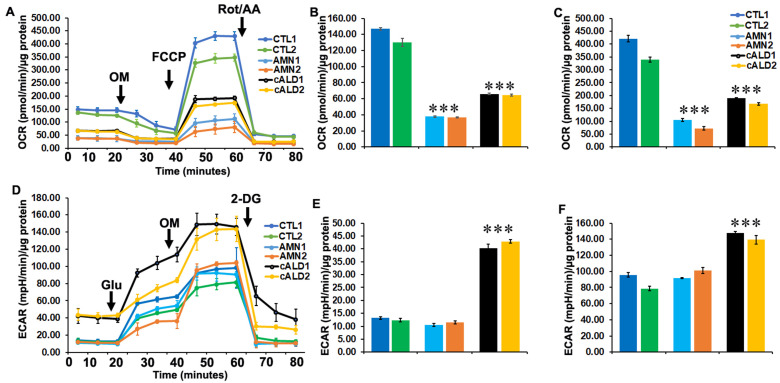
OCR and ECAR measurements in CTL, AMN, and cALD iPSC-derived astrocytes. Results for 1 × 10^5^ cells/well in XF_e_96 V3-PS cell culture microplate (Seahorse Bioscience). (**A**) Representative oxygen consumption rate (OCR) profile of control (CTL), AMN, and ALD iPSC-derived astrocytes. (**B**) Quantitation of basal and (**C**) FCCP-uncoupled OCR. (**D**) Representative ECAR profile of control (CTL), AMN, and ALD iPSC-derived astrocytes. (**E**) Quantitation of basal and (**F**) oligomycin-linked ECAR. Each data point represents mean ± SD (*n* = 6), mpH, milli-pH units. *** *p* < 0.001.

Mitochondrial dysfunction has been reported in X-ALD by us and others. We used a Seahorse XFe96 Analyzer (Seahorse Bioscience, Billerica, MA, USA) to assess the metabolic profile of control healthy, AMN, and cALD patient iPSC-derived astrocytes (Figure 3A). Measurement of oxygen consumption rate (OCR), a measure of oxidative phosphorylation, and extracellular acidification rate (ECAR), a measure of glycolysis, give an overall metabolic status of the cell [12,18]. In line with our recent report [17], control healthy astrocytes from both patients had significantly higher basal OCR while AMN iPSC-derived astrocytes had a significantly lower basal OCR (Figure 3A,B). The basal OCR rate of cALD iPSC-derived astrocytes was higher than in AMN astrocytes but lower than in control astrocytes (Figure 3A,B). The FCCP-uncoupled OCR represents the maximal respiratory capacity of mitochondria and hence is a measure of mitochondrial electron transport chain integrity [12]. The FCCP-uncoupled OCR was most decreased in AMN astrocytes, while cALD astrocytes had levels higher than AMN but lower than control astrocytes (Figure 3A,C).

ECAR levels representing glycolytic rate were significantly higher in cALD astrocytes at basal levels (Figure 3D,E). ECAR levels in response to oligomycin treatment were further increased in cALD astrocytes compared to control and AMN astrocytes (Figure 3D,F).

### 2.8. The Balance of Pro- and Anti-Inflammatory Cytokine Pathway Genes Is Altered Between AMN and cALD Astrocytes

**Figure 4:**
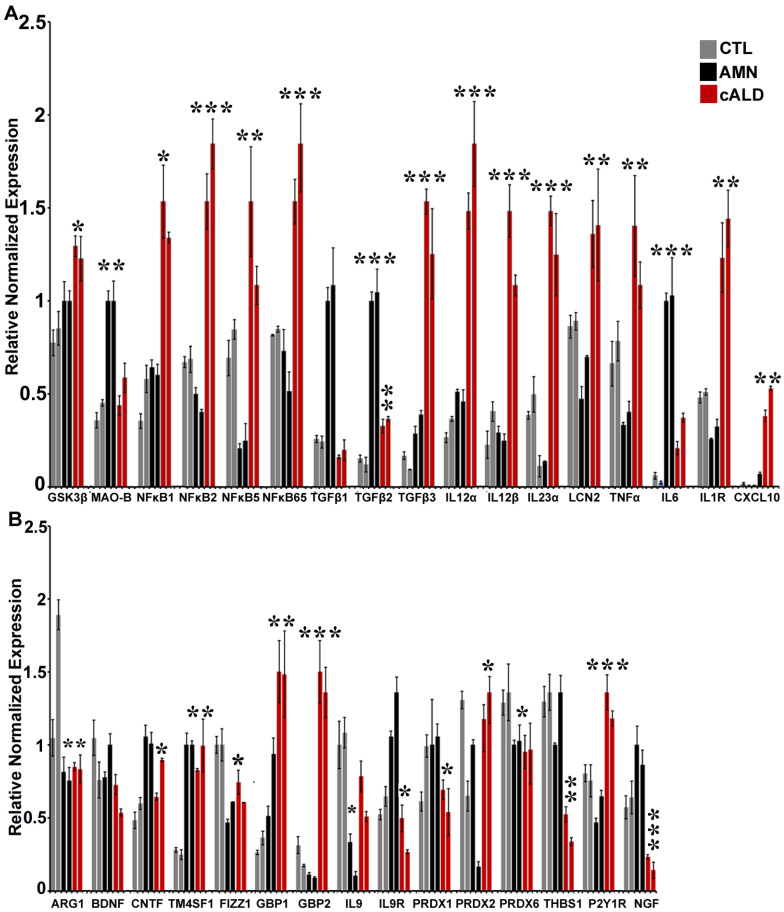
Pro- and anti-inflammatory gene expression. (**A**) Representative proinflammatory and (**B**) anti-inflammatory gene expression in CTL, AMN, and cALD iPSC-derived astrocytes. Values are expressed as mean ± SEM (*n* = 3); * *p* < 0.05, ** *p* < 0.01, *** *p* < 0.001.

Considering that inflammatory response is implicated in the pathophysiology of cALD, we investigated pro- and anti-inflammatory cytokine pathway genes in AMN and cALD iPSC-derived astrocytes. Proinflammatory pathway genes showing differential expression between AMN and cALD astrocytes included GSK3β, MAO-B, NFκB subunits, TGFβ, IL12α, IL12α, IL23α, LCN2, TNFα, IL6, and IL1R (Figure 4A). Gene expressions of GSK3β, NFκB1, NFκB2, NFκB5, NFκB65, TGFβ3, IL12α, IL12β, IL23α, LCN2, TNFα, IL1R, and CXCl10 were significantly increased in cALD astrocytes (Figure 4A), whereas expressions of MAO-B, TGFβ1, TGFβ2, and IL6 were significantly higher in AMN astrocytes (Figure 4A). Among the anti-inflammatory pathway genes measured, expressions of CNTF, IL9R, PRDX1, THBS1, and NGF were significantly decreased in cALD astrocytes compared to AMN astrocytes (Figure 4B). GBP1, GBP2, IL9, PRDX2, and P2Y1R expressions were significantly increased in cALD astrocytes compared with AMN astrocytes (Figure 4B). Gene expressions of ARG1, FIZZ1, and PRDX6 were decreased in both AMN and cALD astrocytes and TM4SF1 was increased in both AMN and cALD astrocytes compared with control astrocytes while there was no significant difference between these genes within AMN compared to cALD astrocytes (Figure 4B).

### 2.9. cALD Astrocytes Have Increased STAT3 Phosphorylation and Decreased AMPK Levels

**Figure 5:**
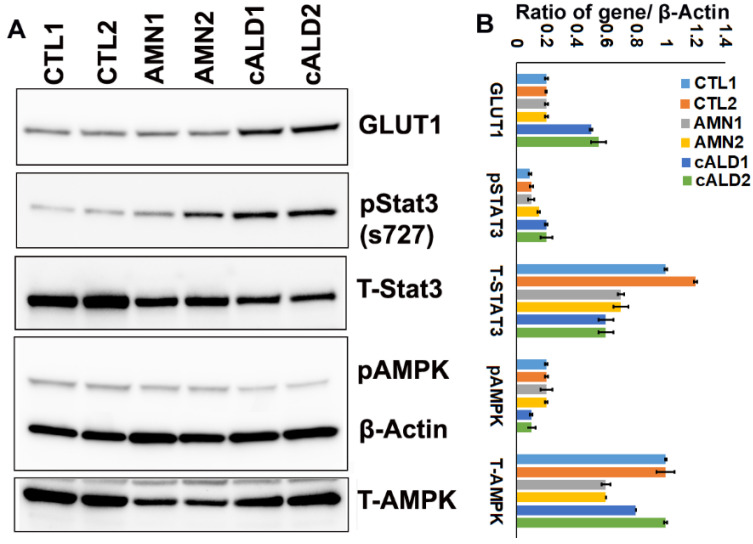
Activation of STAT3 and inhibition of AMPKα1 in cALD astrocytes. (**A**) Western blots to monitor phosphorylation of STAT3 and AMPKα1, and protein content of GLUT1, total STAT3, and AMPKα1 and β-Actin for loading control in CTL, AMN, and cALD astrocytes. (**B**) Histogram of average densitometry data.

To further investigate the inflammatory signaling, we examined phosphorylation and total protein contents of STAT3 and AMPK pathways and GLUT1 levels in control, AMN, and cALD astrocytes (Figure 5). GLUT1 levels were significantly increased in cALD astrocytes compared to AMN and control astrocytes (Figure 5A), corresponding with the increased glycolytic response (ECAR) observed in cALD astrocytes. We previously reported a decrease in AMPK levels in cALD patient post mortem brain and patient-derived cells [12,14,17,18]. Similarly, AMPK phosphorylation at T172 was decreased in cALD astrocytes compared to AMN and control astrocytes (Figure 5A). Ser727 phosphorylation of STAT3 was significantly increased in cALD astrocytes while total STAT3 levels were decreased (Figure 5A). The signal intensities of protein bands in arbitrary units after normalization with the signal intensity of the β-actin internal control were calculated using ImageJ (version 1.54 h) software (Figure 5B).

## 3. Discussion

X-ALD is the most frequent peroxisomal disorder affecting males, yet the disease mechanisms beyond ABCD1 mutation and VLCFA derangement largely remain unknown and there are no satisfactory therapeutic options. A significant barrier in unravelling the disease mechanisms has been lack of a relevant mouse model of the disease. The X-ALD mouse model is a classical knockout of the ABCD1 gene and accumulated VLCFA similar to the human phenotype. It, however, fails to develop CNS demyelination characteristic of the human cALD phenotype. Human iPSC-derived cell models of brain cells provide an opportunity to investigate early events in disease development and also to test novel therapeutic strategies. This study documents iPSC-derived astrocytes generated for apparently healthy controls (CTL), AMN, and cALD phenotypes of X-ALD and adds biological replicates to our recent report [17]. Non-integrating mRNA-based reprogramming used in the present work is advantageous to generate clinical grade stem cells as there is no genome integration of the reprogramming factors. The non-modified mRNA and microRNA technology used to deliver the reprogramming factors (SOX2, LIN28, OCT4, KLF4, NANOG, and MYC) and immune evasion factors allow repeated transfections.

A documented role of astrocyte involvement in X-ALD neuropathology has been provided from human post mortem brain tissue and in vitro mouse astrocyte cultures by our laboratory and others [13,14,18,19,20,21]. Understanding the differential response of astrocytes in AMN and cALD phenotypes has been hampered by the lack of availability of human primary cultures. This has started to change recently with the generation of iPSC-derived brain cells, including astrocytes from AMN and cALD phenotypes [17,22,23]. We recently documented generating astrocytes from iPSC-derived skin fibroblasts of one male patient each of CTL, AMN, and cALD phenotypes [17]. In the present study, we generated and characterized astrocytes obtained from iPSC lines reprogrammed from skin fibroblasts of two additional male patients each of CTL, AMN, and cALD phenotypes. In line with our recent report [17], ABCD1 mutation did not affect the reprogramming ability of AMN and cALD skin fibroblasts and the iPSC lines were successfully differentiated into astrocytes. Immunocytochemical expression analysis of differentiated astrocytes indicated that they were positive for mature astrocyte markers, including GFAP, EAAT, S100β, and ALD1H1, consistent with previous studies [17,22].

Mutations in the ABCD1 gene encoding the ALDP is the primary clinical cause of X-ALD regardless of the phenotype [1]. We observed that, similar to AMN and cALD patient fibroblasts, the ABCD1 expression was lacking in the AMN and cALD iPSC-derived astrocytes. A lack of ALDP is associated with accumulation of VLCFA in patient body fluids and tissues, including the central nervous system [4]. While plasma VLCFA levels do not correlate with disease phenotype variability in male patients, VLCFA is still the only biochemical hallmark of the disease [3,4,6,24]. Mouse astrocytes silenced for Abcd1 and Abcd2 and human transformed cell lines silenced for ABCD1 also accumulate VLCFA [13,21,25]. However, they are unable to account for the differential response seen in AMN and cALD phenotypes. We recently documented differential accumulation of VLCFA in patient iPSC-derived astrocytes from AMN and cALD phenotypes [17]. In line with these, VLCFA levels were also differentially increased in AMN and cALD patient iPSC-derived astrocytes in the present study with higher accumulation in cALD patient iPSC-derived astrocytes. These results also support previous studies showing a higher accumulation of VLCFA in cALD patient iPSC-derived brain cells [22,23].

We and others have previously reported mitochondrial dysfunction in X-ALD [12,14,17,18,22,26]. Mitochondrial function measured as the OCR was significantly lower in AMN astrocytes. The FCCP-uncoupled OCR also represents the maximum respiratory capacity (MRC) of mitochondria and hence is a measure of mitochondrial electron transport chain integrity [24] and was significantly reduced in AMN compared to cALD patient-derived astrocytes. In contrast to the OCR, ECAR levels were significantly higher in cALD patient iPSC-derived astrocytes compared to AMN and control astrocytes. Increased ECAR was in line with the higher GLUT1 protein levels in cALD patient-derived iPSCs. This observation supports our recent report of the association of ECAR and GLUT1 in different cALD patient-derived astrocytes [17]. Higher glycolysis is associated with increased inflammation and mitochondrial dysfunction in glial cells [27,28].

TNFα-stimulated astrocytic STAT3 activation induces vascular inflammation and compromises the BBB [29], underscoring the significance of higher TNFα expression and increased STAT3 phosphorylation observed in cALD astrocytes in this study. The results also support our recent report of increased STAT3 phosphorylation at Ser727 in different cALD patient astrocytes [17]. Though identified originally as a metabolic gene regulating cellular energy homeostasis, increasing evidence implicates the loss of AMPK in spontaneous development of an inflammatory phenotype [30,31,32,33] or a more severe neuroinflammatory response [34]. The activation of AMPK was evaluated by detection of phosphorylation of AMPKα1 (p-AMPKα1) at Thr172. We observed decreased AMPK phosphorylation in cALD iPSC-derived astrocytes in line with our previous reports in the post mortem cALD brain, patient fibroblasts, and cALD patient iPSC-derived astrocytes [12,14,17,18]. The inflammatory phenotype of cALD observed in our study is in keeping with previous reports where decreases in AMPK activity and expression have been shown to result in increased inflammatory response [14,30,31,33,34]. AMPKα1 is crucial for anti-inflammatory skewing of immune cells [30,33,34] and is involved in inhibiting lipid-induced macrophage inflammation [31]. Indeed, AMPK-knockout animal models consistently demonstrate reduced mitochondrial biogenesis/function [35] and increased proinflammatory skewing [30,31,32,33]. We previously reported decreased mitochondrial function (OCR) and increased cytokine response in Abcd1-KO mouse primary astrocytes silenced for AMPKα1 [14].

The role of inflammation and cytokines in X-ALD has been reported by us and others [12,13,14,17,18,36,37,38]. Similar to macrophage polarization, astrocytes are now recognized to have A1 (proinflammatory) and A2 (anti-inflammatory) responses [39]. We investigated this differential response in AMN and cALD patient iPSC-derived astrocytes. Increased cytokine levels have been reported in X-ALD patient plasma, PBMCs, and post mortem brain [12,13,18,19,37,38,40]. We documented increased proinflammatory cytokines in cALD post mortem brain [18], cALD patient lymphocytes [12,14], patient-derived astrocytes [17], and in Abcd1-KO mice astrocytes silenced additionally for Abcd2 [13] or AMPKα1 [14]. Several proinflammatory cytokine genes were differentially upregulated in cALD astrocytes in this study. GSK3β, increased in cALD astrocytes in our study, was previously reported to regulate oxidative stress and inflammatory response in X-ALD patient fibroblasts and Abcd1-KO mice [38]. In line with the role of NFκB in X-ALD inflammatory response reported by us and others [13,17,41], several NFκB subunits had increased expression in cALD astrocytes. TGFβ is a key modulator of astrocyte reactivity and we found higher TGFβ3 expression in cALD astrocytes. Increased TGFβ is also reported in Abcd1-KO mice spinal cord [38]. Increased IL12 levels in cALD astrocytes are supported by previous reports of higher IL12 expression in cALD patient post mortem brain [19] and PBMCs [37]. Increased IL23 levels in cALD astrocytes are in line with a recent report of astrocyte-specific expression of IL23 leading to an enhanced inflammatory response and aggravated phenotype in a multiple sclerosis mouse model [42]. Lipocalin-2 (LCN2) expression reflects the activation state of astrocytes and its levels are increased in inflammatory A1 subtype astrocytes [43]. The expression level of LCN2 is increased through the activation of the NFκB signaling pathway under inflammatory stress conditions [44]. This is in line with the increased LCN2 observed in cALD astrocytes in this study and increased NFκB signaling in mouse astrocytes silenced for Abcd1/Abcd2 and in patient iPSC-derived astrocytes [13,17]. Increased IL6 and TNFα expression have been reported in X-ALD plasma [40], PBMCs [37] and post mortem brain [19]. Interestingly, increased IL6 levels in in AMN and decreased levels in cALD astrocytes observed by us are similar to the plasma IL6 profiles of AMN and cALD reported earlier [37]. Increased IL1 levels observed in cALD astrocytes our study are supported by previous reports from cALD post mortem brain [19]. The chemokine CXCL10, increased in cALD astrocytes, is expressed in neuronal cells and is implicated in neurodegenerative and neuroinflammatory responses and the influx of inflammatory leukocytes into neural tissue. CXCL10 is upregulated early in MS and mainly released by astrocytes [45].

Several anti-inflammatory genes were differentially expressed between AMN and cALD astrocytes. ARG1 and FIZZ1, considered to be neuroprotective [46], were decreased in AMN and cALD astrocytes. Astrocyte neurotrophic factors such as BDNF, CNTF, and NGF are implicated in the support of neuronal survival. Interestingly, BDNF inhibited GSK3β which in turn inhibits NFκB signaling and proinflammatory cytokines [47]. CNTF, an important promyelinating factor, inhibited inflammatory pathology in experimental allergic encephalomyelitis (EAE), a mouse model of multiple sclerosis [48] and astrocyte-derived CNTF protected oligodendrocytes from TNFα insult [49]. NGF, via its high-affinity receptor TrkA, exerted an anti-inflammatory effect by inhibiting GSK3β activity, reducing IκB phosphorylation and p65 NFκB translocation, and increasing nuclear p50 NFκB binding activity [50]. The guanine-binding protein (GBP) family ranges in size from 65 to 67 kDa and includes two members in humans, GBP1 and GBP2 [51]. GBP1 was induced in human stimulated astrocytes [52] and GBP2 is considered indicative of inflammatory A1 astrocytes [53]. Thus, downregulation of GBPs is indicative of the A2 astrocyte phenotype and supports our observation of increased GBP1 and GBP2 expression in cALD astrocytes. While IL9 was originally described as a growth factor from the Th9 subtype of T-cells [54], its receptor, IL9R, is found on astrocytes and oligodendrocytes [55]. Neutralization of IL9 ameliorates EAE [56] and is consistent with increased expression of IL9 seen in cALD astrocytes. Peroxiredoxin (PRDX) represents a family of antioxidant enzymes that play a crucial role in preventing oxidative damage. PRDX1 expression in astrocytes is considered neuroprotective in the CNS [57,58] and PRDX2 correlated with the degree of oxidative stress and inflammation in MS lesions [59]. PRDX6 was identified as an important factor regulating the response of astrocytes towards Aβ plaques and astrocyte/microglia crosstalk [60].

Thrombospondins (THBS1 and THBS2), a novel family of astrocyte-secreted proteins, promote the formation of excitatory synapses [61]. Increased astrocytic THBS activity is considered an effective approach to prevent depression [62] and enhanced THBS1 is proposed to be a compensatory mechanism for controlling immune response and protecting tissue for excessive damage [63,64]. THBS1 expression was significantly decreased in cALD astrocytes. The P2Y1 receptor (P2Y1R) is involved in over-activation of astrocytes in an Alzheimer’s disease (AD) mouse model and may correlate with the progression of Alzheimer’s disease [65]. Silencing P2Y1R in astrocytes protected against neuroinflammation and cognitive decline in an AD mouse model [66].

In summary, iPSC-derived astrocytes from AMN and cALD male patients in the present study demonstrate differential biochemical, mitochondrial, and cytokine responses representative of two major X-ALD phenotypes. Combining the observations in iPSC-derived astrocytes from the present study together with our recent report [17], these patient-derived cell models can be a useful tool for mechanistic studies into differential inflammatory responses of astrocytes in AMN and cALD phenotypes. We recognize the limitation that the cultured astrocytes utilized in the present study represent a simplified model relative to that of astrocytes in the CNS. However, our human astrocyte culture model offers a unique system to delineate the autonomic responses of AMN and cALD astrocytes. Future studies could investigate these responses in co-culture systems of astrocytes with neurons and/or oligodendrocytes. Additionally, a multi-omics approach could provide valuable insight into broader changes occurring in disease-relevant X-ALD patient iPSC-derived astrocytes and other cell types, potentially identifying novel targets for therapeutic interventions. Given that X-ALD mouse models do not develop the neuroinflammatory phenotype, cALD iPSC-derived astrocytes represent a valuable model as a screening tool for the development of novel therapeutic strategies.

## 4. Material and Methods

### 4.1. Ethics Approval

The study protocol was approved by the IRB (#13352). Fibroblast samples were de-identified specimens obtained from Coriell Cell repositories and did not involve recruitment of human subjects.

### 4.2. Human Fibroblasts

Healthy human skin CTL fibroblasts (GM08402; 32-year-old male, GM03348; 10-year-old male), AMN fibroblasts (GM17819; 32-year-old male, GM07675; 22-year-old male), and cALD fibroblasts (GM04904; 11-year-old male, GM04496; 6-year-old male) were obtained from the National Institute for General Medical Sciences human genetic cell repository at Coriell Institute for Medical Research, Camden, NJ, USA.

### 4.3. Derivation of iPSCs and Differentiation into Astrocytes

iPSCs were generated from skin fibroblasts with the Stemgent^®^ StemRNA™ 3rd Gen Reprogramming Kit (Reprocell). The iPSCs were functionally characterized by checking for their ability to differentiate into the three germ layers (Human pluripotent stem cell functional identification kit, R&D systems, Cat#SC027B) according to the manufacturer’s protocol. Neural precursor cells (NSCs) were generated from human induced pluripotent stem cells (iPSCs) using a STEMdiff^TM^ SMADi Neural Induction kit (STEMCELL Technologies) via dual SMAD inhibition as per the manufacturer’s protocol. The NPCs were differentiated with a STEMdiff^TM^ Astrocyte differentiation kit and STEMdiff^TM^ Astrocyte maturation kit (STEMCELL Technologies) as per the manufacturer’s protocol.

### 4.4. Mycoplasma Detection

The presence of mycoplasma was checked by PCR using a mycoplasma detection kit (Venor^®^ GeM OneStep, Minerva biolabs, Berlin, Germany).

### 4.5. Culturing of Mature iPSC-Derived Astrocytes

Astrocytes were cultured at a seeding density of 5 × 10^4^ cells/cm^2^ (day 0) at 37 °C and 5% CO_2_ in astrocyte medium (Neurobasal-A medium, Thermo Fisher Scientific, Waltham, MA, USA) containing N21 max (1X, R&D Systems, Minneapolis, MN, USA), FBS One Shot (1X, Thermo Fisher Scientific), Glutamax (1X, Gibco, Thermo Fisher Scientific), heregulin-β1 (10 ng/mL, Peprotech, Inc., Cranbury, NJ, USA), bFGF (8 ng/mL, R&D Systems), and penicillin–streptomycin (1X, ThermoFisher Scientific, Waltham, MA, USA).

### 4.6. VLCFA Analysis

CTL, AMN, and cALD astrocytes (2.5 × 10^5^ cells each) were processed at the Wayne State University Lipidomics Core facility. Saturated hexacosanoic acid (C26:0) level was calculated per microgram of protein. Lipids were subject to alkaline methanolysis and the resulting fatty acid methyl esters were analyzed by gas chromatography–mass spectrophotometry (QP2010 GC-MS system, Shimadzu Scientific Instruments, Kyoto, Japan) equipped with a Restek column, as reported [5].

### 4.7. Mitochondrial Oxygen Consumption and Glycolytic Function Measurement

Oxygen consumption rate (OCR) and extracellular acidification rate (ECAR) were measured using a Seahorse Bioscience XFe96 Extracellular Flux Analyzer, as described previously [12].

### 4.8. Quantitative Real-Time Polymerase Chain Reaction Gene Expression

Total RNA was extracted with the miRNeasy kit (Qiagen) and 1 µg RNA was used for cDNA synthesis. RT-qPCRs were conducted using a CFX96 Real-Time PCR Detection System (BioRad, Hercules, CA, USA) using IQ SYBR Green Supermix (BioRad), as described previously [17]. Gene expression was normalized to the 60 S ribosomal L27 gene and samples were run in triplicate. Primer sequences of the genes investigated are listed in Appendix A.

### 4.9. Immunofluorescence Staining

CTL, AMN, and cALD cells were plated on Matrigel-coated chambered slides (25,000 cells/cm^2^) and allowed to grow overnight. Cells were fixed with 4% paraformaldehyde for 15 min at room temperature (RT), washed with PBS, and incubated in blocking solution containing 10% normal donkey serum (Sigma) and 0.03% Triton X-100 (Sigma-Aldrich, St. Louis, MO, USA) in PBS for 1 h at RT. Primary antibodies were incubated overnight at 4 °C and secondary antibodies for 1 h at RT. Nuclei were counterstained with DAPI (Sigma). Images were acquired with a fluorescence microscope (BZ-X series, Keyence, Osaka, Japan). The antibodies used are listed in Appendix B.

### 4.10. Western Blot Analysis

CTL, AMN, and cALD astrocytes were homogenized in radioimmunoprecipitation (RIPA) buffer with protease inhibitor cocktail (Thermo Fisher Scientific). Then, 60 μg of total protein was electrophoresed as described previously [17]. The antibodies used are listed in Appendix B.

### 4.11. Karyotyping

The iPSCs (2 × 10^6^ cells) at passage 5 were analyzed using a KaryoStat^TM^ assay (ThermoFisher Scientific).

### 4.12. Data Analysis

Data were analyzed using GraphPad Prism software (version 7.0). Normality was assessed with the Kolmogorov–Smirnov test. Groups were compared with a two-tailed unpaired Student’s *t*-test for normally distributed data or a non-parametric Mann–Whitney test for non-normally distributed data. Statistical significance was set at *p* < 0.05.

## Data Availability

The original contributions presented in this study are included in the article/Supplementary Material. Further inquiries can be directed to the corresponding author.

## Supplementary Materials

The following supporting information can be downloaded at: https://www.mdpi.com/article/10.3390/ijms26041576/s1.

## Appendix A. List of Human Real-Time Primers Used

**Name**	**Sequence**
OCT4-F	GAAACCCACACTGCAGATCA
OCT4-R	GGTTACAGAACCACACTCG
NANOG-F	AGATGCCTCACACGGAGACT
NANOG-R	TTTGCGACACTCTTCTCTGC
SOX2-F	TGCTGCCTCTTTAAGACTAGGAC
SOX2-R	CCTGGGGCTCAAACTTCTCT
LIN28-F	GGCAGTGGAGTTCACCTTTAAGA
LIN28-R	AGCTTGCATTCCTTGGCATGATGA
L27-F	TGGACAAAACTGTCGTCAATAAGG
L27-R	AGAACCACTTGTTCTTGCCTGTC
TM4SF1-F	GGCTACTGTGTCATTGTGGCAG
TM4SF1-R	ACTCGGACCATGTGGAGGTATC
THBS1-F	GCTGGAAATGTGGTGCTTGTCC
THBS1-R	CTCCATTGTGGTTGAAGCAGGC
THBS2-F	CAGTCTGAGCAAGTGTGACACC
THBS2-R	TTGCAGAGACGGATGCGTGTGA
IL9-F	GACCAGTTGTCTCTGTTTGGGC
IL9-R	TTTCACCCGACTGAAAATCAGTGG
IL-9R-F	ATCAGTCCTGCCTTGGAGCCAA
IL-9R-R	CCGACAATGTGATCCCTGTGCT
MAO-F	GTGAAGCAGTGTGGAGGCACAA
MAO-R	TTCACTCGGTCTCCAAGGAGGT
NFkB1-F	GCAGCACTACTTCTTGACCACC
NFkB1-R	TCTGCTCCTGAGCATTGACGTC
NFkB2-F	GGCAGACCAGTGTCATTGAGCA
NFkB2-R	CAGCAGAAAGCTCACCACACTC
NFkB65-F	TGAACCGAAACTCTGGCAGCTG
NFkB65-R	CATCAGCTTGCGAAAAGGAGCC
GBP1-F	TAGCAGACTTCTGTTCCTACATCT
GBP1-R	CCACTGCTGATGGCATTGACGT
GBP2-F	GTTCCTACATCCTCAGCCATTCC
GBP2-R	CCACTGCTGATGGCATTGACGT
PRDX1-F	CTGCCAAGTGATTGGTGCTTCTG
PRDX1-R	AATGGTGCGCTTCGGGTCTGAT
PRDX2-F	CCTTCCAGTACACAGACGAGCA
PRDX2-R	CTCACTATCCGTTAGCCAGCCT
PRDX6-F	CAGCTACCACTGGCAGGAACTT
PRDX6-R	GGAAGGACCATCACACTATCCC
LCN2-F	GTGAGCACCAACTACAACCAGC
LCN2-R	GTTCCGAAGTCAGCTCCTTGGT
TGFb1-F	TACCTGAACCCGTGTTGCTCTC
TGFb1-R	GTTGCTGAGGTATCGCCAGGAA
TGFb2-F	AAGAAGCGTGCTTTGGATGCGG
TGFb2-R	ATGCTCCAGCACAGAAGTTGGC
TGFb3-F	CTAAGCGGAATGAGCAGAGGATC
TGFb3-R	TCTCAACAGCCACTCACGCACA
GLUT1-F	TTGCAGGCTTCTCCAACTGGAC
GLUT1-R	CAGAACCAGGAGCACAGTGAAG
IL12α-F	TGCCTTCACCACTCCCAAAACC
IL12α-R	CAATCTCTTCAGAAGTGCAAGGG
IL12β-F	GACATTCTGCGTTCAGGTCCAG
IL12β-R	CATTTTTGCGGCAGATGACCGTG
IL23α-F	GAGCCTTCTCTGCTCCCTGATA
IL23α-R	GACTGAGGCTTGGAATCTGCTG
IL1R-F	GTGCTTTGGTACAGGGATTCCTG
IL1R-R	CACAGTCAGAGGTAGACCCTTC
TNFα-F	CTCTTCTGCCTGCTGCACTTTG
TNFα-R	ATGGGCTACAGGCTTGTCACTC
ARG1-F	TCATCTGGGTGGATGCTCACAC
ARG1-R	GAGAATCCTGGCACATCGGGAA
FIZZ1-F	GCAAGAAGCTCTCGTGTGCTAG
FIZZ1-R	AACATCCCACGAACCACAGCCA
BDNF-F	CATCCGAGGACAAGGTGGCTTG
BDNF-R	GCCGAACTTTCTGGTCCTCATC
CNTF-F	TCAGACCTGACTGCTCTTACGG
CNTF-R	TTGGAGTCGCTCTGCCTCGGT
NGF-F	ACCCGCAACATTACTGTGGACC
NGF-R	GACCTCGAAGTCCAGATCCTGA
P2RY1-F	GCCATCTGGATGTTCGTCTTCC
P2RY1-R	TGGCAGAGTCAGCACGTACAAG

## Appendix B. List of Antibodies Used

**Catalog No**	**Antibody Details**
5685S	EAAT1 (D20D5) RABBIT mAb
59678S	AQP4(D1F8E) XP RABBIT mAb
85828S	ALDH1L1(E712Q) RABBIT mAb
G3893	MONOCLONAL ANTI-GLIAL FIBRILLARY ACID PROTEIN (GFAP)
S2532	MONOCLONAL ANTI-S-100B
AB53521–1001	Anti-A2B5 antibody
5568S	BETA-3-TUBULIN
5332S	Desmin (D93F5) XP^®^ Rabbit mAb
963121	Goat anti-human SOX17
963273	Goat anti-human Otx2
963427	Goat anti-human Brachyury
73349S	NESTIN
AF2018	Human/Mouse/Rat SOX2 Affinity Purified Polyclonal Ab
60064AD	Anti-Human TRA-1–60 Antibody, Clone TRA-1–60R, Alexa Fluor^®^ 488
60062PE	Anti-Human SSEA-4 Antibody, Clone MC-813–70, PE
2840S	Oct-4A Rabbit mAb
AF1997	Human Nanog Antibody
MAB2018	Human/mouse/rat Sox2
2840S	Oct-4A Rabbit mAb
AF-1759	Human/Mouse/Oct-3/4 Antibody
23064S	Sox2 (D9B8N) Rabbit mAb
ab197013	ABCD1/ALD antibody [EPR15929]
**Secondary Antibodies**	
A21206	Alexa Fluor 488 Donkey Anti Rabbit
A21042	Alexa Fluor 488 Goat anti mouse
A11055	Alexa Fluor 488 Donkey anti goat
705–605–147	Alexa Fluor 647 Donkey Anti Goat
715–605–150	Alexa Fluor 647 Donkey Anti mouse
711–605–152	Alexa Fluor 647 Donkey Anti Rabbit
**Manufacturer**	**Dilution**
CELL SIGNALING	1/200
CELL SIGNALING	1/400
CELL SIGNALING	2/100
SIGMA	1/100
SIGMA	1/100
ABCAM	1/100
Cell Signaling	1/200
Cell Signaling	1/100
R&D	1/100
R&D	1/100
R&D	1/100
CELL SIGNALING	1/400
R&D	5 µg/mL
STEM CELL TECH	2/100
STEM CELL TECH	2/100
Cell Signaling	1/100
R&D	10 µg/mL
R&D	8 µg/mL
Cell Signaling	1/100
R&D	10 µg/mL
Cell Signaling	1/100
Abcam	1/1000
Invitrogen	1/200
Invitrogen	1/200
Invitrogen	1/200
JacksonImmunoResearch	1.5/100
JacksonImmunoResearch	1.5/100
JacksonImmunoResearch	1.5/100
