# Supplementary material for: Generation and Characterization of Human iPSC-Derived Astrocytes with Potential for Modeling X-Linked Adrenoleukodystrophy Phenotypes
Source: Int J Mol Sci. 2025 Feb 13;26(4):1576. doi: 10.3390/ijms26041576 (PMC11855073; doi:10.3390/ijms26041576)
Supplement: Supplementary file 1 [file ijms-26-01576-s001.zip › ijms-3415644-supplementary.pdf]

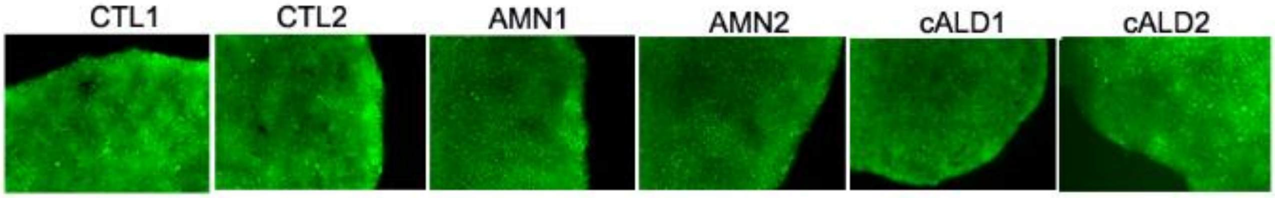

Supplementary Figure S1: Alkaline phosphatase live staining. Magnification 10X

Thermo Fisher SCIENTIFIC

Supplementary Figure S2

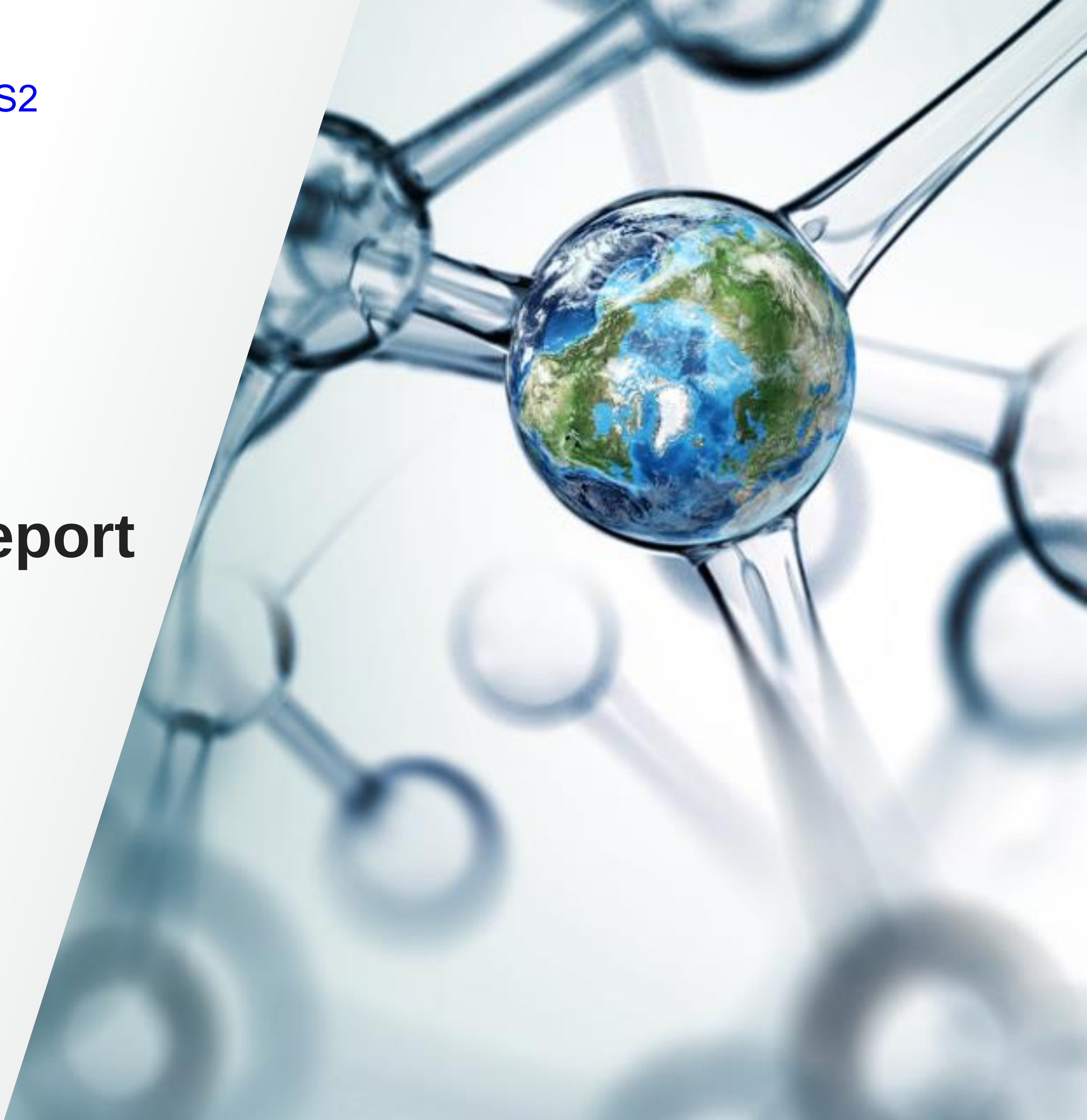

KaryoStat+ ™ and Cell ID ™ Report

Client Name: **Henry Ford Health System**
Quote No: **10745555**
Prepared by: **Kevin J. Velez**

The world leader in serving science

Summary of Services

Thermo Fisher SCIENTIFIC

Project Summary:

- Henry Ford Health System is interested in services provided by the Life Technologies Corporation in the analysis of twelve (12) client-provided samples using the KaryoStat+ and Cell ID assay.

Service Description:

- The KaryoStat™ assay allows for digital visualization of chromosome aberrations with a resolution similar to g-banding karyotyping. The size of structural aberration that can be detected is > 2 Mb for chromosomal gains and > 1 Mb for chromosomal losses (the resolution depends on the location of the aberration in the chromosome. Due to a lower probe density on the telomere ends and centromeres, the resolution in those locations may be closer to 5Mb). The KaryoStat array is optimized for balanced whole-genome coverage with a low-resolution DNA copy number analysis, the assay covers all 36,000 RefSeq genes, including 14,000 OMIM® targets. The assay enables the detection of aneuploidies, submicroscopic aberrations, and mosaic events.

- Using the same array as the Karyostat Assay, the Cell ID assay allows for DNA fingerprint matching of human cell lines through correlation analysis of 1.1M SNPs between samples. The detection of 1.1M SNPs across the genome allows for unique DNA based signatures of the genetic background of a cell, which can then be compared with others.

Methods:

1. Genomic DNA purification:
   - Cells were prepared according to the PureLink™ Genomic DNA Purification Kit (Catalog #: K1820-02) and quantified using the NanoDrop ONE^{C} (Catalog # : 701-058108).

2. GeneArray® Preparation:
   - 100 ng total gDNA was used to prepare the GeneArray® for KaryoStat according to the manual, and is an array that looks for SNPs, copy number variants and single nucleotide polymorphisms across the genome.

Sample Information

#	Sample ID	Status
KS-17415	7	Complete
KS-17416	8	Complete
KS-17417	9	Complete
KS-17418	10	Complete
KS-17419	11	Complete
KS-17420	12	Complete

Table S1: Karyostat⁺ Sample ID. Customer provided sample information

KaryoStat+ Results: KS-17415

1. KaryoStat+ analysis of this sample revealed that it originated from a male individual.
2. No chromosomal aberrations were found when comparing against the reference dataset.

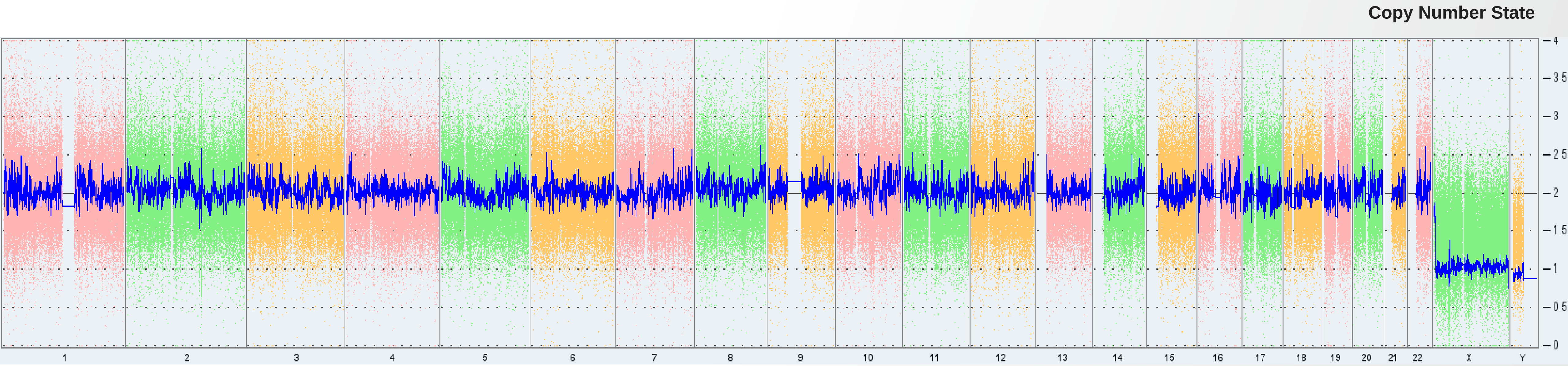

Figure 6: Whole genome view. The whole genome view displays all somatic and sex chromosomes in one frame with high level copy number. The smooth signal plot (right y-axis) is the smoothing of the log2 ratios which depict the signal intensities of probes on the microarray. A value of 2 represents a normal copy number state (CN = 2). A value of 3 represents chromosomal gain (CN = 3). A value of 1 represents a chromosomal loss (CN = 1). The pink, green and yellow colors indicate the raw signal for each individual chromosome probe, while the blue signal represents the normalized probe signal which is used to identify copy number and aberrations (if any). Aberrations when present are indicated by red arrows.

Disclaimer: This assay was conducted solely for the listed investigator/institution. The results of this assay are for research use only.

KaryoStat+ Results: KS-17416

1. KaryoStat+ analysis of this sample revealed that it originated from a male individual.
2. No chromosomal aberrations were found when comparing against the reference dataset.

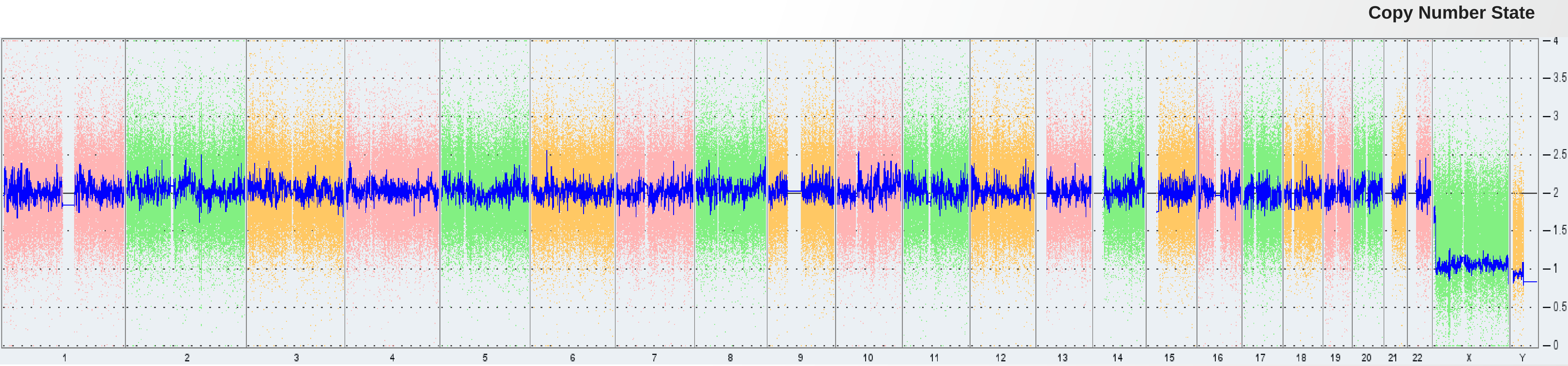

Figure 7: Whole genome view. The whole genome view displays all somatic and sex chromosomes in one frame with high level copy number. The smooth signal plot (right y-axis) is the smoothing of the log2 ratios which depict the signal intensities of probes on the microarray. A value of 2 represents a normal copy number state (CN = 2). A value of 3 represents chromosomal gain (CN = 3). A value of 1 represents a chromosomal loss (CN = 1). The pink, green and yellow colors indicate the raw signal for each individual chromosome probe, while the blue signal represents the normalized probe signal which is used to identify copy number and aberrations (if any). Aberrations when present are indicated by red arrows.

Disclaimer: This assay was conducted solely for the listed investigator/institution. The results of this assay are for research use only.

KaryoStat+ Results: KS-17417

1. KaryoStat+ analysis of this sample revealed that it originated from a male individual.
2. No chromosomal aberrations were found when comparing against the reference dataset.

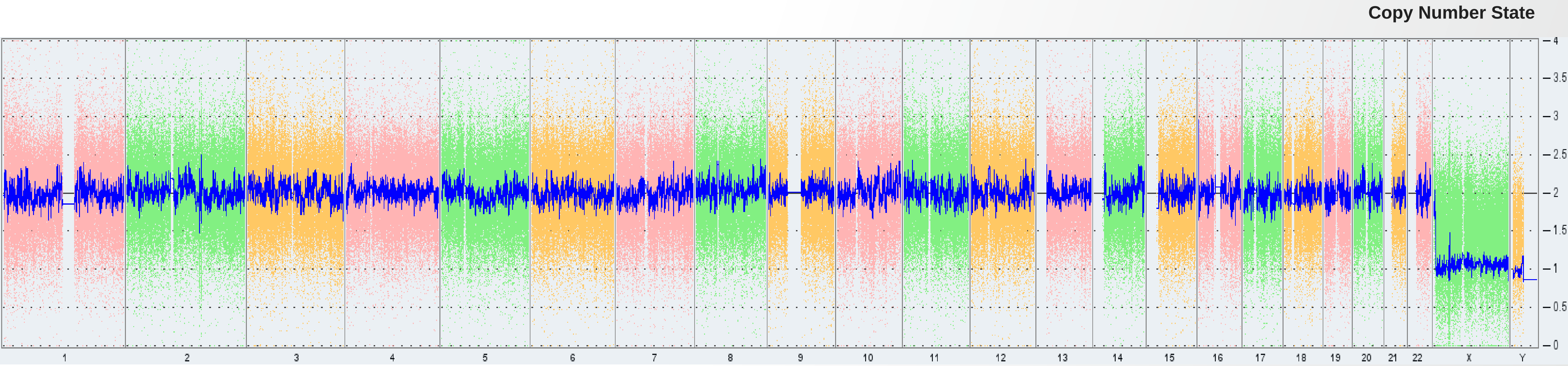

Figure 8: Whole genome view. The whole genome view displays all somatic and sex chromosomes in one frame with high level copy number. The smooth signal plot (right y-axis) is the smoothing of the log2 ratios which depict the signal intensities of probes on the microarray. A value of 2 represents a normal copy number state (CN = 2). A value of 3 represents chromosomal gain (CN = 3). A value of 1 represents a chromosomal loss (CN = 1). The pink, green and yellow colors indicate the raw signal for each individual chromosome probe, while the blue signal represents the normalized probe signal which is used to identify copy number and aberrations (if any). Aberrations when present are indicated by red arrows.

Disclaimer: This assay was conducted solely for the listed investigator/institution. The results of this assay are for research use only.

KaryoStat+ Results: KS-17418

1. KaryoStat+ analysis of this sample revealed that it originated from a male individual.
2. No chromosomal aberrations were found when comparing against the reference dataset.

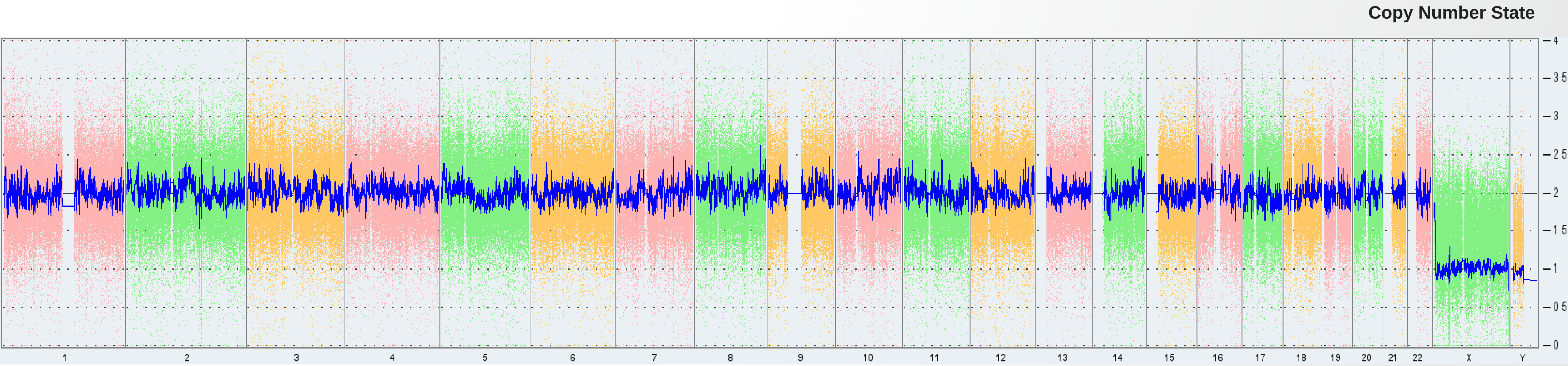

Figure 9: Whole genome view. The whole genome view displays all somatic and sex chromosomes in one frame with high level copy number. The smooth signal plot (right y-axis) is the smoothing of the log2 ratios which depict the signal intensities of probes on the microarray. A value of 2 represents a normal copy number state (CN = 2). A value of 3 represents chromosomal gain (CN = 3). A value of 1 represents a chromosomal loss (CN = 1). The pink, green and yellow colors indicate the raw signal for each individual chromosome probe, while the blue signal represents the normalized probe signal which is used to identify copy number and aberrations (if any). Aberrations when present are indicated by red arrows.

Disclaimer: This assay was conducted solely for the listed investigator/institution. The results of this assay are for research use only.

KaryoStat+ Results: KS-17419

1. KaryoStat+ analysis of this sample revealed that it originated from a male individual.
2. No chromosomal aberrations were found when comparing against the reference dataset.

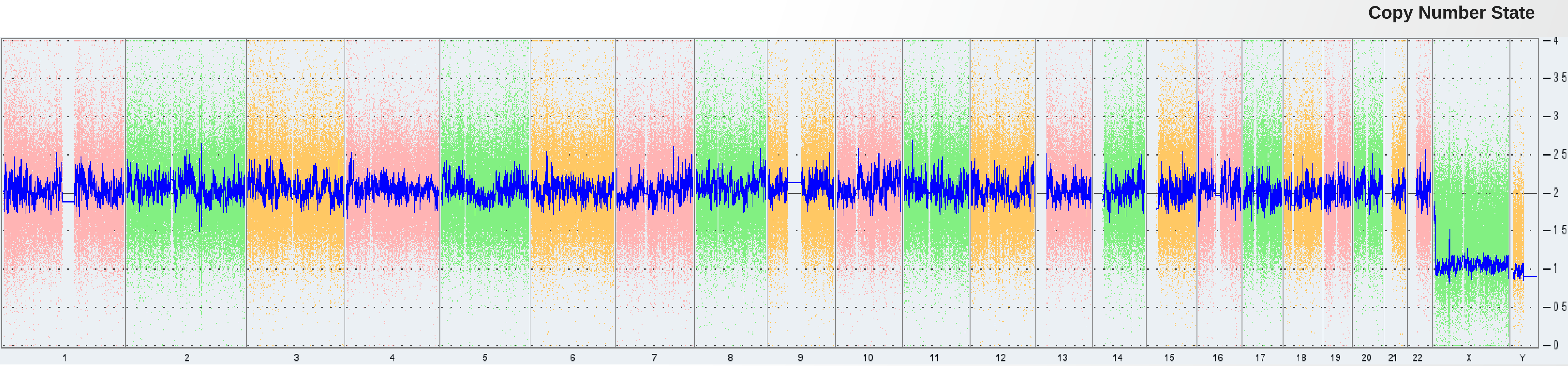

Figure 10: Whole genome view. The whole genome view displays all somatic and sex chromosomes in one frame with high level copy number. The smooth signal plot (right y-axis) is the smoothing of the log2 ratios which depict the signal intensities of probes on the microarray. A value of 2 represents a normal copy number state (CN = 2). A value of 3 represents chromosomal gain (CN = 3). A value of 1 represents a chromosomal loss (CN = 1). The pink, green and yellow colors indicate the raw signal for each individual chromosome probe, while the blue signal represents the normalized probe signal which is used to identify copy number and aberrations (if any). Aberrations when present are indicated by red arrows.

Disclaimer: This assay was conducted solely for the listed investigator/institution. The results of this assay are for research use only.

KaryoStat+ Results: KS-17420

1. KaryoStat+ analysis of this sample revealed that it originated from a male individual.
2. No chromosomal aberrations were found when comparing against the reference dataset.

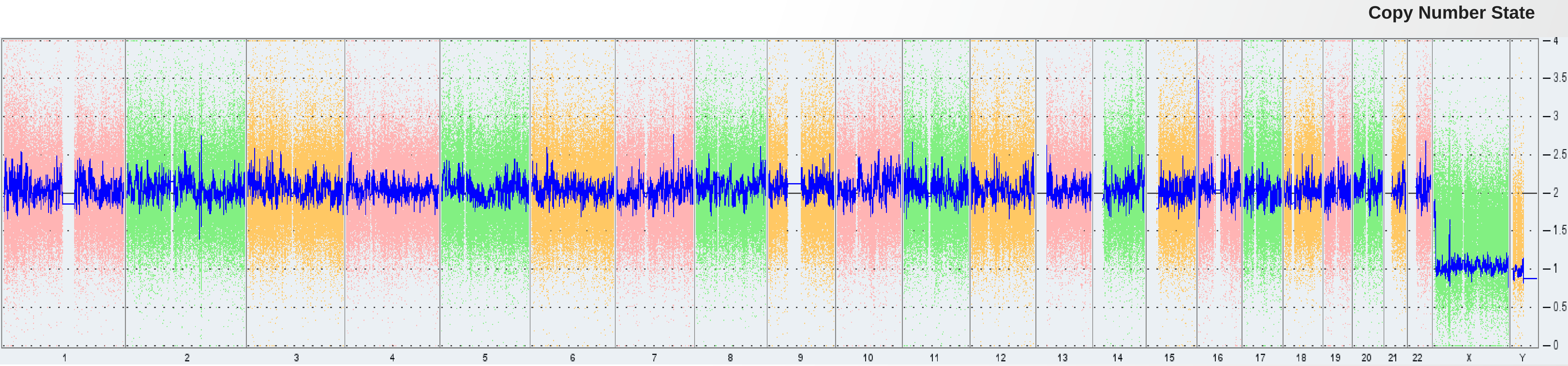

Figure 11: Whole genome view. The whole genome view displays all somatic and sex chromosomes in one frame with high level copy number. The smooth signal plot (right y-axis) is the smoothing of the log2 ratios which depict the signal intensities of probes on the microarray. A value of 2 represents a normal copy number state (CN = 2). A value of 3 represents chromosomal gain (CN = 3). A value of 1 represents a chromosomal loss (CN = 1). The pink, green and yellow colors indicate the raw signal for each individual chromosome probe, while the blue signal represents the normalized probe signal which is used to identify copy number and aberrations (if any). Aberrations when present are indicated by red arrows.

Disclaimer: This assay was conducted solely for the listed investigator/institution. The results of this assay are for research use only.

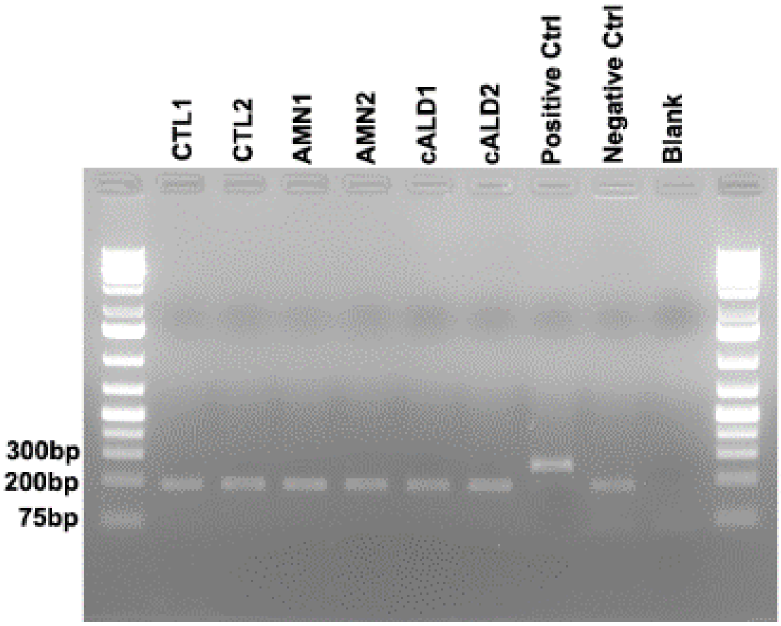

Supplementary Figure S3: Mycoplasma detection

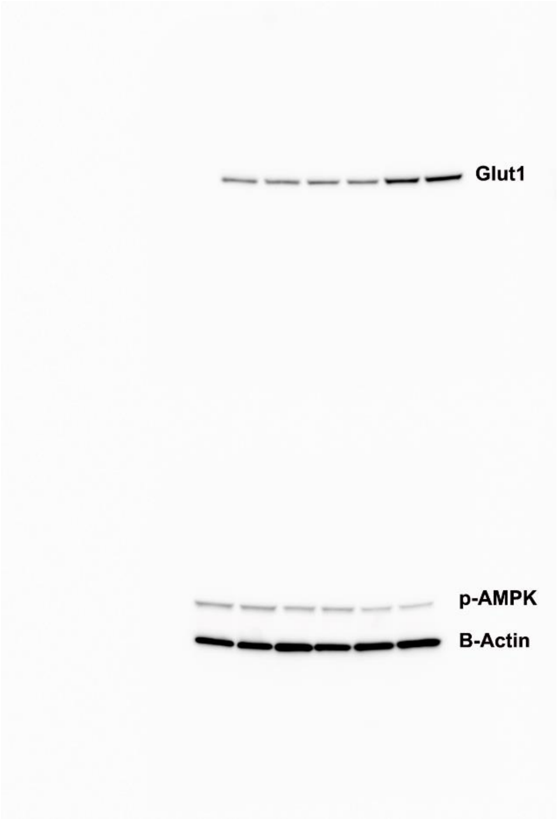

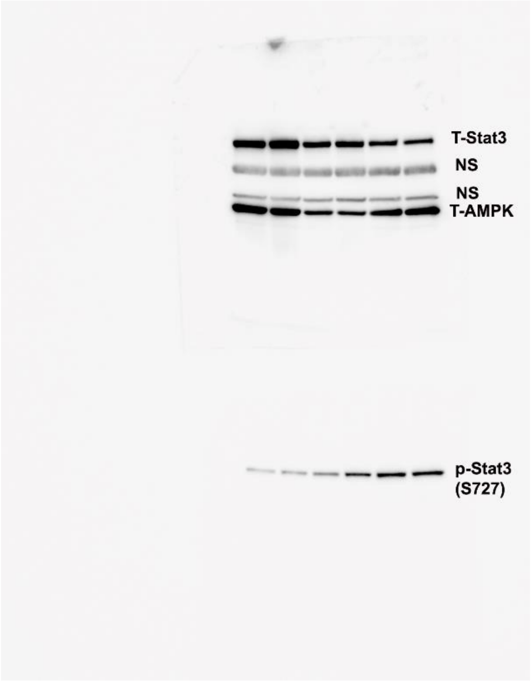

Supplementary Figure S4: Original western blots for Glut 1, pStat3, pAMPK, T-Stat3 and T-AMPK.
